# The Venoarteriolar Reflex Significantly Reduces Contralateral Perfusion as Part of the Lower Limb Circulatory Homeostasis *in vivo*

**DOI:** 10.3389/fphys.2018.01123

**Published:** 2018-08-17

**Authors:** Henrique Silva, Hugo A. Ferreira, Hugo P. da Silva, L. Monteiro Rodrigues

**Affiliations:** ^1^Research Center for Biosciences and Health Technologies, Universidade Lusófona's CBiOS, Lisbon, Portugal; ^2^Pharmacological Sciences Department, Faculty of Pharmacy, Universidade de Lisboa, Lisbon, Portugal; ^3^Faculty of Sciences, Institute of Biophysics and Biomedical Engineering, Universidade de Lisboa, Lisbon, Portugal; ^4^IT - Instituto de Telecomunicações, Lisbon, Portugal; ^5^School of Technology, Polytechnic Institute of Setúbal, Setúbal, Portugal

**Keywords:** venoarteriolar reflex, photoplethysmography, wavelet transform, vascular regulation, flowmotion analysis

## Abstract

Perfusion at microvascular level involves the contribution of both local and central regulators, under a complex vascular signaling frame. The venoarteriolar reflex (VAR) is one of such regulatory responses, of particular relevance in the lower limb to prevent edema. Although known for quite some time, many of the complex interactions involving all of these regulatory mechanisms still need clarification. Our objective was to look deeper into VAR through modern photoplethymography (PPG). Twelve healthy subjects (both sexes, 26.0 ± 5.0 y.o.) were enrolled in this study after informed written consent. Subjects were submitted to a leg lowering maneuver while lying supine to evoke the VAR, involving three phases–10 min baseline register, both legs extended, 10 min challenge, with one randomly chosen leg (test) pending 50 cm below heart level, while the contralateral (control) remained in place, and 10 minutes recovery, resuming the initial position. PPG signals were collected from both feet and treated by the wavelet transform (WT) revealing six spectral bands in frequency intervals comprising the cardiac [1.6–0.7 Hz], respiratory [0.4–0.26 Hz], myogenic [0.26–0.1 Hz], neurogenic/sympathetic [0.1–0.045 Hz], endothelial NO-dependent (NOd) [0.045–0.015 Hz], and NO-independent (NOi) [0.015–0.007 Hz] activities. For the first time, this approach revealed that, with VAR, perfusion significantly decreased in both limbs, although the change was more pronounced in the test foot. Here, a significant decrease in myogenic, neurogenic and NOd, were noted, while the control foot recorded a decrease in neurogenic and an increase in NOd. These results confirm the utility of WT spectral analysis for flowmotion. Further, it strongly suggests that VAR results from a complex cooperation between local myogenic-endothelial responses, where a central neurogenic reflex might also be involved.

## Introduction

Microcirculation is critical for nutrient delivery, also for waste removal, contributing to peripheral vascular resistance through various complex mechanisms acting on local Starling forces (Levy et al., 2001). Microcirculation encompasses the smallest diameter blood vessels (<150 μm) in the cardiovascular system, capable to respond to local perfusion changes through variations of its vascular wall tone (Jonk et al., 2007) induced by local or central reflexes. One important vascular response for the maintenance of stable blood flow to peripheral tissues is the venoarteriolar reflex (VAR), also known as postural vasoconstrition reflex. The VAR refers to the reduction in limb blood perfusion in the dependent position due to an increase in pre-capillary vascular resistance. Changing the limb position immediately reduces blood perfusion (~40%) by increasing venous transmural pressure, which evokes arteriolar vasoconstriction (Hassan and Tooke, 1988; Rathbun et al., 2008). This arteriolar vasoconstriction, in turn, decreases regional blood flow and reduces capillary pressure, protecting the dependent limb against blood accumulation and edema (Gabrielsen and Norsk, 2007). An impaired venoarterial reflex may explain swelling in patients with metabolic impairment (e.g., diabetics) or in special physiological periods (e.g., edema during the luteal phase of menstrual cycle) (Rayman et al., 1986; Hassan et al., 1990).

There is a great deal of conflicting data and uncertainty regarding VAR mechanisms. It is generally accepted that VAR is a purely axonal reflex since it requires an intact neuronal network and can be abolished by blocking sympathetic nervous transmission (Henriksen and Sejrsen, 1976; Henriksen, 1977; Hassan and Tooke, 1988; Vissing et al., 1997). However, there is no agreement about the involved neural pathway. It has been previously suggested that postganglionic sympathetic axons had the dual role of sensing venous distension and, in turn, inducing arteriolar vasoconstriction. For this reason, VAR was also called the “local axon response,” with the central nervous system not being necessary to evoke the response (Henriksen, 1977; Moy et al., 1989). Recent evidence has suggested that VAR does not occur through α-adrenergic mechanisms, but rather by a non-adrenergic neural mechanism (Crandall et al., 2002). A possible contribution of a myogenic mechanism associated to changes in vascular pressure has also been proposed (Crandall et al., 2002; Okazaki et al., 2005; Gabrielsen and Norsk, 2007).

Most of these findings were obtained by non-invasive technologies, in particular, Laser Doppler flowmetry (LDF) and Photoplethysmography (PPG) using the skin as a convenient vascular network (Holowatz et al., 2008). In fact, skin microvascular function is generally regarded as an experimental mirror of microvascular beds (Jung et al., 2001; Shamim-Uizzaman et al., 2002; Rossi et al., 2009) including cardiac muscle. However, signal analysis from these instruments, based in similar (light transmission) biophysics, offers multiple difficulties, due to their oscillatory characteristics (Sahni, 2012; Silva et al., 2015).

Our main objective in this study is to look deeper into the physiological mechanism involved in the vascular response to a classic VAR by a tested experimental setup that simplifies procedures, reduces variability sources (Rathbun et al., 2008; Silva et al., 2015; Rocha et al., 2017), using modern PPG, and explores new analytical directions through the wavelet transform.

## Materials and methods

### Volunteers

Twelve healthy, non-smoking subjects (26.0 ± 5.0 y.o., seven females, five males) participated in this study after informed written consent. Procedures respected all principles of good clinical practice determined by the Declaration of Helsinki and subsequent amendments (World Medical Association, 2013). Consumption of caffeine-containing foods and beverages 24 h prior to the conduction of experiments was an imposed restraint.

### Experimental

PPG is a long known, simple to use, low-cost optical technique applied to quantitatively describe (in arbitrary units) the microcirculation perfusion conditions *in vivo* (Allen, 2007). Its basic principle, related with LDF, quantifies the amount of visible light reflected by microcirculatory vessels, which is in proportion to the circulating blood volume. The PPG waveform is composed of alternating current (AC, or pulsatile) and direct current (DC, or steady) components. The DC component corresponds to the detected optical signal from the tissue and depends on the structure of the tissue and the average blood volume of both arterial and venous blood. The AC component relates to the changes in blood volume created by arterial pulses occurring between the systolic and diastolic phases of the cardiac cycle. It has been recently proposed that the modulation of PPG light intensity may be attributed to the compression/decompression of the capillary bed caused by varying arterial transmural pressure (Kamshilin et al., 2015), but also to changes in RBCs orientation in the capillaries (Volkov et al., 2017). Thus, PPG waveform shares similar biophysics with LDF being probably determined by the same components as recent data seems to suggest (Silva et al., 2017). Its waveform, therefore, shares some optical basis with LDF, being probably affected by the same components already identified for that technique.

Recent technological advances, introducing new semiconductor components, portability and computer-based pulse wave analysis renewed the interest for this technique in recent years (Allen, 2007; Silva et al., 2016; Kamshilin and Margaryants, 2017), opened new directions while preserving a consistent low cost availability. Our PPG system uses a 520 nm light wavelength providing a signal relatively free of artifacts, reaching less than 0.6 mm depth, compatible with the measurement of the dermal capillary loops on the dermal papillae (Braverman, 2000; Kamshilin and Margaryants, 2017).

**Figure 1:**
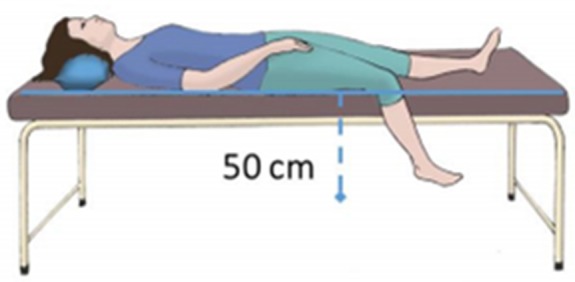
Illustrative scheme of the posture adopted by the subject during the challenge phase of the protocol.

Acclimatization took place with individuals lying supine in a room with controlled temperature (22 ± 1°C) and humidity (40–60%). A standard VAR protocol (Rathbun et al., 2008) was set in place, consisting of three phases—a 10 min baseline recording phase, in a supine position with both legs lying parallel to the body axis (Phase I – baseline); a 10 min recording phase with one randomly chosen leg pending approximately 50 cm below the thigh (Phase II – challenge) (Figure 1); a final 10 min register in the initial position (Phase III – recovery). The lower limb perfusion changes were assessed by reflection PPG (PLUX Wireless Biosignals, Portugal) including a blood volume pressure sensor connected to a BITalino Plugged board (PLUX Wireless Biosignals, Portugal). Two probes were applied on the plantar surface of the first toe in both feet, one serving as the test (T), and the contralateral as the control (C). Signals were acquired at a 100 Hz sampling rate, and expressed in terms of arbitrary units (AU). Blood perfusion was expressed as the amplitude of the PPG waveform.

### Analytical

PPG signals were imported into MATLAB (Mathworks R2012, USA), for further processing. Raw signals were first smoothed with a moving average filter, downsampled to 32Hz, and then decomposed into their main oscillatory components using a wavelet transform (WT) toolbox (http://noc.ac.uk/using-science/crosswavelet-wavelet-coherence).

WT analysis is a well-known analysis tool for refinement. A wavelet is a small wave or oscillation that decays quickly. Wavelets are considered a family of functions constructed from translations and dilations of a single function called the “mother wavelet” ψ(t) (Chuang et al., 2013). They are defined by:

(1) $$\begin{array}{l} \psi_{\text{a},\text{b}}\left(\text{t}\right)=\frac{1}{\sqrt{\left|\text{a}\right|}}\psi \left(\frac{\text{t}-\text{b}}{\text{a}}\right),\text{ a},\text{b}\in \text{R},\text{ a}\neq 0 \end{array}$$

The parameter a is the scaling parameter or scale, and it measures the degree of compression. The parameter b is the translation parameter which determines the time location of the wavelet. If |a| < 1, then the wavelet in the above equation is the compressed version (smaller support in time-domain) of the mother wavelet and corresponds mainly to higher frequencies. On the other hand, when |a| > 1, then ψ_a,b_(t) has a larger time-width than ψ(t) and corresponds to lower frequencies. Thus, wavelets have time-widths adapted to their frequencies (Sifuzzaman et al., 2012). WT analysis allows the decomposition of complex signals possessing several length scales into their main frequency components and the estimation of the contribution of each component to the overall signal in each time point (Bracic and Stefanovska, 1998).

PPG signals seem to display activity components similar to those known for LDF in the same frequency ranges (Mizeva et al., 2015; Silva et al., 2017). Each component's activity was described as the percent ratio of its area under the curve (AUC) on the signal frequency spectrum to the AUC of the entire spectrum. For statistical purposes, three analysis periods within each of the recording times were used lying inside the WT cone of influence (COI) – Phase I (4:00 min to 9:00 min), Phase II (12:00 min to 17:00 min) and Phase III (21:00 min to 26:00 min). PPG amplitude and the component's ratios were compared between phases within each foot with the Wilcoxon signed rank test, and between feet with the Mann-Whitney *U*-test. For all tests, a *p* < 0.05 was adopted.

## Results

Table 1 summarizes the perfusion and the components' amplitude ratio values for both feet in baseline, challenge and recovery phases of all subjects. Comparing values from men and women revealed no differences between sexes in any of the experimental phases. The spectral analysis of the PPG signals revealed similar frequency intervals for the six components previously described for LDF (Figure 4).

**Table 1:** Mean ± sd of the PPG amplitude and components' amplitude ratios for each phase of the protocol (C, control foot; T, test foot).

	**Test foot**			**Control foot**			**p (baseline, C vs. T)**	**p (challenge, C vs. T)**	**p (recovery, C vs. T)**
	**Baseline**	**Challenge**	**Recovery**	**Baseline**	**Challenge**	**Recovery**			
PPG amplitude (AU)	123.6 ± 69.5	15.3 ± 14.2	62.7 ± 29.3	154.5 ± 120.9	106.4 ± 89.7	107.7 ± 102.0	0.320	0.010^*^	0.040^*^
	–	0.010^*^	0.003^*^	–	0.018^*^	0.003^*^			
Cardiac (%)	19.6 ± 11.3	3.7 ± 3.8	10.0 ± 5.7	22.5 ± 13.6	18.2 ± 18.2	16.4 ± 16.4	0.514	<0.001^*^	0.178
	–	0.002^*^	0.002^*^	–	0.010^*^	0.003^*^			
Respiratory (%)	3.7 ± 1.9	3.2 ± 2.4	6.8 ± 5.3	2.6 ± 0.9	3.2 ± 1.4	3.2 ± 1.7	0.128	0.478	<0.001^*^
	–	0.182	0.020^*^	–	0.012^*^	0.077			
Myogenic (%)	14.0 ± 8.4	7.9 ± 5.1	14.0 ± 7.0	11.8 ± 5.6	11.3 ± 6.4	15.3 ± 7.4	0.630	0.060	0.630
	–	0.023^*^	0.969	–	0.182	0.008^*^			
Sympathetic (%)	11.1 ± 4.5	6.4 ± 3.4	21.1 ± 4.6	15.5 ± 5.4	14.5 ± 4.4	15.3 ± 5.3	0.095	<0.001^*^	0.020^*^
	–	0.013^*^	0.003^*^	–	0.011^*^	0.050			
Endothelial NO-dependent (%)	10.2 ± 4.3	6.1 ± 2.5	10.5 ± 3.4	14.5 ± 4.8	25.4 ± 9.8	17.9 ± 6.7	0.069	<0.001^*^	0.004^*^
	–	0.028^*^	0.530	–	0.003^*^	0.583			
Endothelial NO-independent (%)	11.0 ± 4.6	14.4 ± 2.9	6.8 ± 2.5	12.7 ± 6.3	12.0 ± 5.7	16.6 ± 7.4	0.671	0.178	<0.001^*^
	–	0.012^*^	0.010^*^	–	0.423	0.006^*^			

Statistical comparison with baseline for each foot, and comparison between feet (

* *p < 0.05)*.

During the challenge phase, perfusion in the test foot significantly decreased (*p* = 0.010), together with several other components' activities, namely the cardiac (*p* = 0.002), myogenic (*p* = 0.023), neurogenic/sympathetic (*p* = 0.013), NOd (*p* = 0.028) and NOi (*p* = 0.012). After returning to the initial position (recovery phase), perfusion was still significantly lower than baseline (*p* = 0.003). The cardiac (*p* = 0.002) and NOi (*p* = 0.010) activities were also found to be significantly lower than baseline, whereas the respiratory (*p* = 0.020) and sympathetic (*p* = 0.003) were found to be significantly higher.

**Figure 2:**
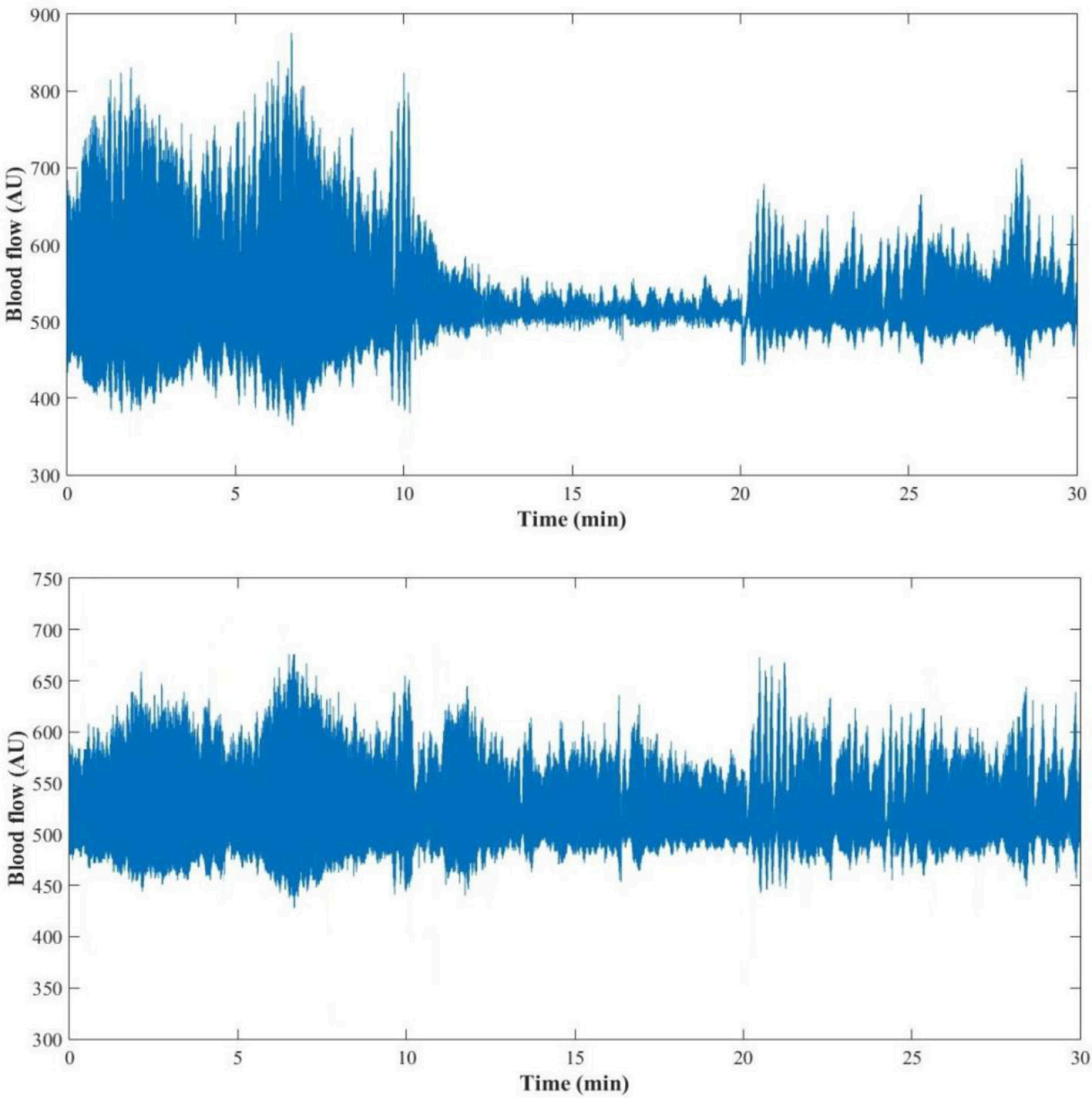
PPG signal of one representative subject (female, 29 years old) during the 30 min (baseline: 0–10 min; challenge: 11–20 min; recovery: 21–30 min) protocol for the test **(Top)** and the control foot **(Bottom)**.

Interestingly, at the contralateral foot perfusion also decreased significantly (*p* = 0.018), during the challenge phase, although the decrease was less pronounced (Figure 2). The cardiac (*p* = 0.010) and neurogenic (*p* = 0.011) activities decreased significantly, while the respiratory (*p* = 0.012) and NOd (*p* = 0.003) increased significantly. In the recovery phase, perfusion was still significantly lower than baseline (*p* = 0.003), as the cardiac component (*p* = 0.003). The myogenic (*p* = 0.008) and NOi (*p* = 0.006) activities were significantly higher during recovery in comparison to baseline. The mean time evolution of the PPG components between the control and test feet is shown in Figure 5. No significant differences were found between feet during baseline. During the challenge, the perfusion of the lowered foot was significantly lower than the control foot (*p* = 0.010). The cardiac and neurogenic activities decreased in both feet (*p* < 0.001 for both cases), but again the effect was more pronounced in the test foot. The NOd (*p* < 0.001) activity was significantly different between feet, having decreased in the test foot but increased in the control. During recovery, the perfusion of both feet was still significantly different (*p* = 0.040), as were the respiratory (*p* < 0.001), neurogenic (*p* = 0.020), NOd (*p* = 0.004) and NOi (*p* < 0.001) activities.

## Discussion

The rhythmic oscillation of vasculature diameter, the vasomotion, described for more than 150 years (Jones, 1852) results in periodic oscillations (Aalkjær et al., 2011) or flowmotion, which actually represent the influence of heartbeat, respiration, myogenic, neurogenic and endothelial activities (Bollinger et al., 1993; Kvernmo et al., 1999; Söderström et al., 2003; Kvandal et al., 2006; Bruning et al., 2015). The mechanisms that produce vasomotion and its physiological purpose are still not clear. However, its consequence, the flowmotion also called skin flowmotion, can be measured. These activities were first described by laser Doppler flowmetry (LDF) signals, and respective frequency ranges were identified (Landsverk et al., 2007). Fourier spectral analysis and Power Spectrum Densities (PSD) have been used to look for conditions associated with alteration of resting skin blood flow, flowmotion, and endothelial dysfunction, justifying the use of spectral analysis as a non-invasive technique to study vasoreactivity. Nevertheless, Fast Fourier analysis is limited by the low resolution capacity for low frequency phenomena, a difficulty that is easily overcome with the Wavelet Transform (Beck et al., 2005).

**Figure 3:**
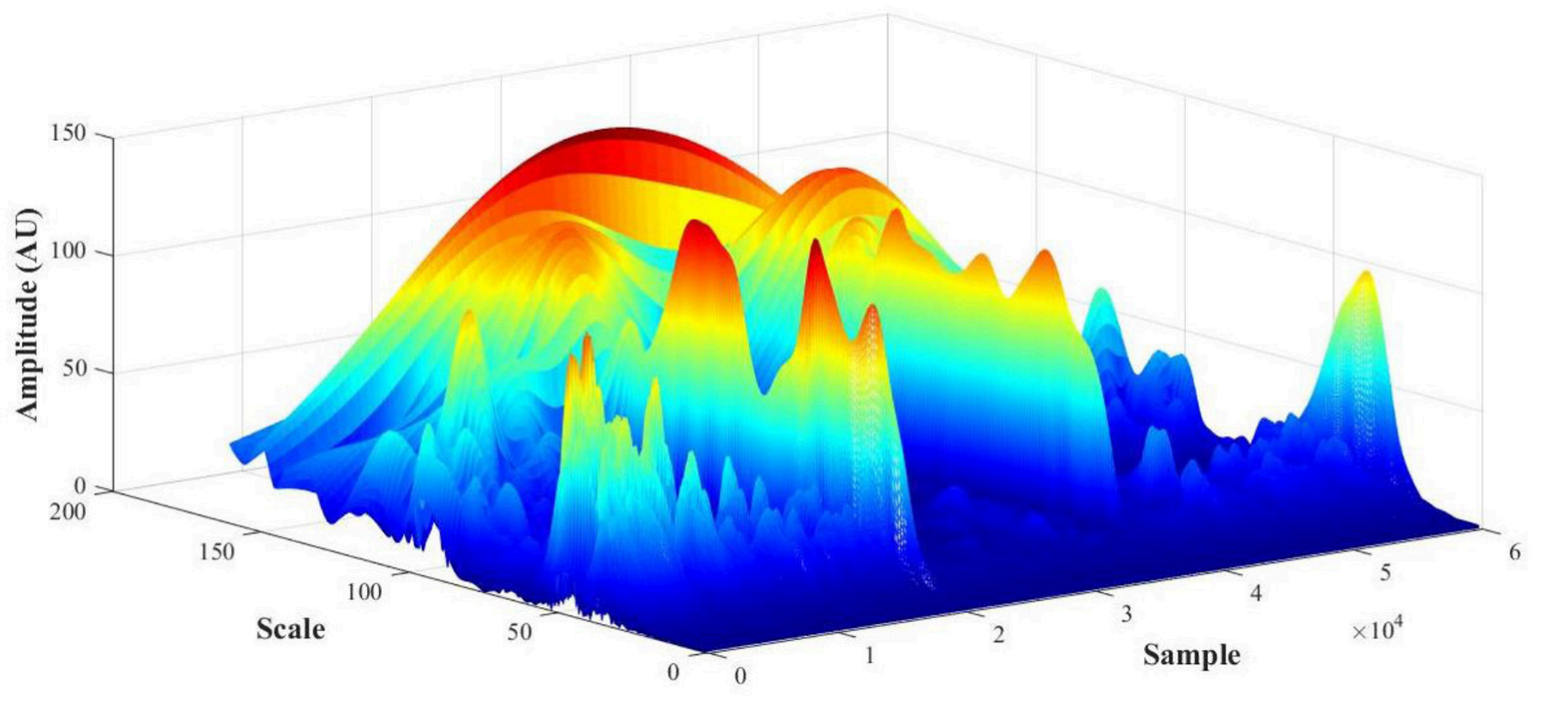
PPG scalogram (scale vs. sample vs. amplitude) for a representative subject (female, 29 y.o., test foot).

**Figure 4:**
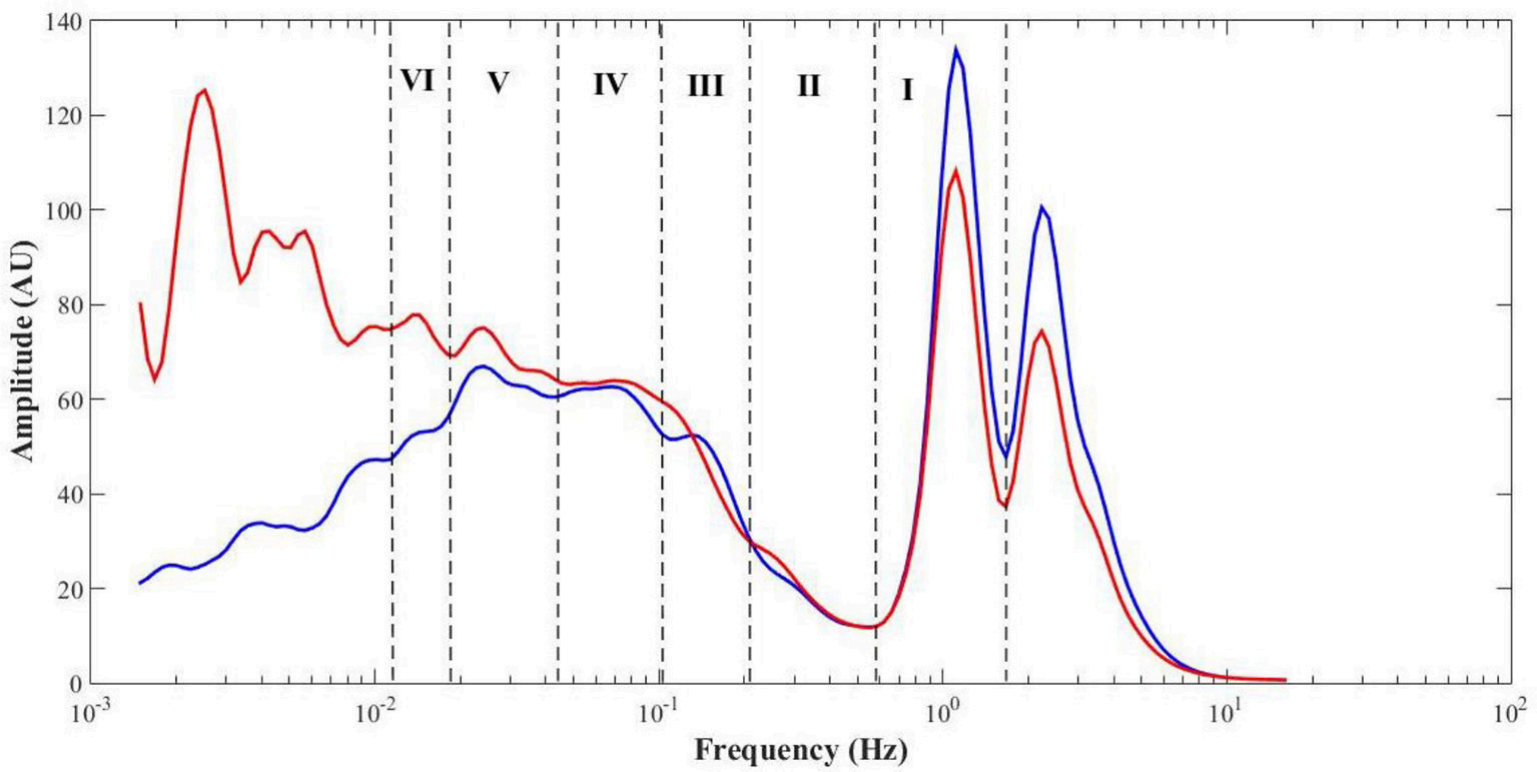
Mean (all phases, *N* = 12) PPG frequency spectra from the test (red) and control (blue) feet, with highlight to the cardiac (I), respiratory (II), myogenic (III), sympathetic (IV), endothelial NO-dependent (V), and endothelial NO-independent (VI) spectral regions.

PPG recordings (expressed in arbitrary flow units AU) were converted to frequency spectra from the WT in MATLAB and the amplitude (also in AU) calculated to detect cardiac [1.6–0.7 Hz], respiratory [0.4–0.26 Hz], myogenic [0.26–0.1 Hz], neurogenic/sympathetic [0.1–0.045 Hz], endothelial NO-dependent (NOd) [0.045–0.015 Hz] and NO independent (NOi) [0.015–0.007 Hz] activities (Mizeva et al., 2015; Silva et al., 2017). For each PPG signal, the initial output of the WT is a 3D scalogram (Figure 3), a signal sample vs. scale vs. amplitude plot. From this representation, the frequency spectrum was constructed by averaging the absolute wavelet transform amplitude at each scale (now converted to frequency) and presenting it in a 2D plot, one axis being the scale (frequency), the other axis being the averaged amplitude (Figure 4). This wavelet-derived frequency spectrum is analogous to the PSD provided by the fast Fourier transform but giving smoother plots which greatly simplify the analysis.

**Figure 5:**
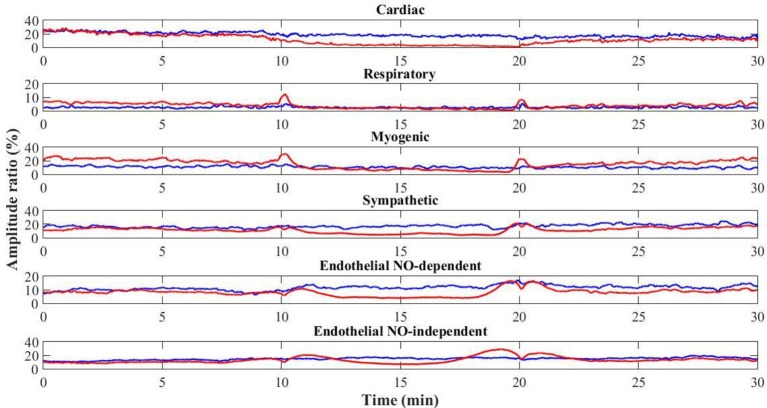
Time evolution of the mean (*N* = 12) PPG signal components for the control (blue) and test (red) feet throughout the protocol (baseline: 0–10 min; challenge: 11–20 min; recovery: 21–30 min).

No differences in perfusion between feet were detected in the baseline phase (Table 1). High levels of estrogen might affect skin flowmotion, but all our female volunteers were in the second phase of their sexual cycles, not taking any contraceptive medicines meaning we can assume a similarly consistent and stable perfusion in both limbs for all volunteers. By placing the test foot in a dependent position, blood accumulated on the extremity, increasing both the arterial hydrostatic and venous pressures in those vascular territories. The increase in venous pressure triggered the VAR which significantly decreased skin perfusion. The observed decrease in the cardiac activity (Table 1, Figure 4) results from the decrease in the transmission of blood pressure oscillations to the peripheral circulation and is therefore expressed by the decrease of the PPG waveform amplitude. These overall changes in myogenic, neurogenic and endothelial activities involved a change in the local vascular regulation dynamics. The increase of the NOi activity together with the decrease in the sympathetic activity could be attributed to the PPG amplitude decrease. The decrease in myogenic activity could be a direct response to the increase in vascular transmural pressures, following foot dependency, as has been previously proposed (Crandall et al., 2002; Okazaki et al., 2005; Krupatkin, 2009).

It has been demonstrated that perivascular adrenergic fibers are sensitive to changes in local transmural pressures. Short term changes occurring with muscle stretching can increase sympathetic-mediated vasoconstriction through activation of vascular α-adrenoreceptors (Haug et al., 2003), whereas long-term changes in the load sustained by a limb can decrease that same activity via a decrease in perivascular nerve density (Zhang, 2001). Our results indicate a decrease in the neurogenic component. The decrease in NOd and NOi activities might be related with a reduction of the release of endothelial mediators resulting from the perfusion reduction. When the perfusion pressure decreases, the endothelial sheer stress also decreases, which lowers the release of vasodilators, NO and prostaglandins in particular (Huang et al., 1995; Sun et al., 1999). Thus, according to this analysis, the decrease in skin perfusion of the dependent foot seems to result from the combined effects on local activities changes. The decrease in vasomotion amplitude and the decrease in endothelial vasodilators release are believed to cause a decrease in perfusion, overcoming the eventual decrease in sympathetic vasoconstrictor activity. Thus, our analysis seems to be in line with previous reports (Crandall et al., 2002; Okazaki et al., 2005; Gabrielsen and Norsk, 2007) relating VAR with an intense local cooperation between myogenic and endothelial activities that persists during recovery.

Interestingly, analysis obtained in the contralateral non-dependent limb offered a completely different view. As far as our knowledge goes, this is the first time the VAR reflex is described by the skin flowmotion from both limbs, showing basically the same trend for the vasomotion components along time, although with some differences (Figure 4). A concomitant perfusion decrease, together with a decrease in myogenic and neurogenic activities, is obvious, although less pronounced than in the depending limb. The decrease in cardiac activity is again related with decreasing blood pressure oscillations transmission to the peripheral circulation, which in turn could be explained by a vasodilation evoked by the decrease in sympathetic activity and the increase in NOd. Here, the respiratory activity responded contrary to the test foot. This is not uncommon and might result from spectral interference of the myogenic activity since both are partially overlapped in the frequency spectrum (Figure 4) (Bollinger et al., 1993). Nevertheless, this common perfusion response registered in both limbs, and in particular, in the resting foot after evoking the VAR in the opposite foot does suggest a centrally-mediated neurogenic response. Recent studies applying this methodology, reported similar phenomena in human (Rocha et al., 2017, 2018) as in mice (Monteiro Rodrigues et al., 2018). Although we cannot conclude that this response is adrenergic in nature or not, it is likely evoked by the abruptly intense myogenic and endothelial cooperation registered in the depending limb and is a response to regulate the regional circulation physiology. The complex relationship between endothelial, myogenic and neurogenic activities has become increasingly evident (Silber et al., 2001; Sandoo et al., 2010; Bruno et al., 2012), and these results may be a different expression of that cooperation, now brought to light by the WT spectral decomposition of PPG perfusion signals from both limbs during VAR.

## Conclusions

Our results describe, for the first time, an effective impact of VAR in the contralateral limb, corroborated by other observations about a wider regional control in the lower limb perfusion homeostasis. Furthermore, the flowmotion analysis provided by the WT of PPG signals seems to offer a better time resolution and smoother frequency spectra, especially for low frequency phenomena, allowing a new insight into the involved mechanisms. The initial reflex in the depending limb, explained by a myogenic-endothelial cooperation, seems now to be only a part of the mechanism. An additional control ruling the regional perfusion appears to be present and will be further investigated.

## Ethics statement

This study was carried out in accordance with the recommendations of Ethical Principles for Medical Research Involving Human Subjects, Declaration of Helsinki. The protocol was approved by the School of Health Sciences and Technologies' Ethical Commission. All subjects gave written informed consent in accordance with the Declaration of Helsinki.

## Author contributions

HS and LM conceived the original idea. HS carried out data collection. HS and HF performed data processing. HS and LM wrote the manuscript. All authors discussed the results and contributed to the final manuscript.

### Conflict of interest statement

The authors declare that the research was conducted in the absence of any commercial or financial relationships that could be construed as a potential conflict of interest.
